# Divergent Giant Clam Strategies to Cope with Thermal Stress Revealed by Fatty Acid Dynamics

**DOI:** 10.1021/acs.est.6c03210

**Published:** 2026-07-02

**Authors:** Isis Guibert, Leonard Pons, Taihun Kim, Jane Ching Yan Wong, Ava Yee Tung Chan, Patrick Cabaitan, Cecilia Conaco, Jetty Chung-Yung Lee, David Michael Baker

**Affiliations:** † Swire Institute of Marine Science, 25809 The University of Hong Kong, Hong Kong SAR 999077, China; ‡ School of Life Sciences, Simon F.S. Li Marine Science Laboratory, The Chinese University of Hong Kong, Hong Kong SAR 999077, China; § Korea Institute of Ocean Science & Technology, Tropical and Sub-Tropical Research Center, Jeju-do 63349, Republic of Korea; ∥ Marine Science Institute, University of the Philippines Diliman Quezon City 1101, Philippines

**Keywords:** giant clams, thermal stress, Symbiodiniaceae, fatty acids, total lipid, bleaching, coral reef, holobiont

## Abstract

Giant clams are mixotrophic bivalves that obtain food from filtration and photosynthetic *Symbiodiniaceae* algae, making them vulnerable to climate change, as elevated sea temperatures trigger bleaching. While the species-specific reliance of giant clams on autotrophy has been reported, their metabolic responses to thermal stress remain poorly understood. Differences in symbiont density and photoefficiency, as well as total lipid and fatty acid (FA) composition of *Tridacna derasa* and *Tridacna maxima* and respective symbionts, highlighted varying abilities to cope with stress during a 31 day thermal stress regime, followed by an 8 day recovery period. *T. derasa* lost 56% of its symbionts, while *T. maxima* lost 89% but nearly recovered. The FA composition was altered in both species, emphasizing the importance of symbiont photosynthate transfers (e.g., C18:2n-6) and suggesting oxidative damage. In addition, FAs exhibited species-specific differences in intensity and rate (e.g., C20:5n-3). Our study is the first to demonstrate the importance of giant clam symbionts in providing essential FAs to their hosts. Our findings highlight significant differences in how species respond to thermal stress, with *T. derasa* exhibiting a resistance strategy and *T. maxima* exhibiting a resilience strategy. This is critical knowledge to understand the biological responses of these species and to inform future conservation strategies.

## Graphical abstract

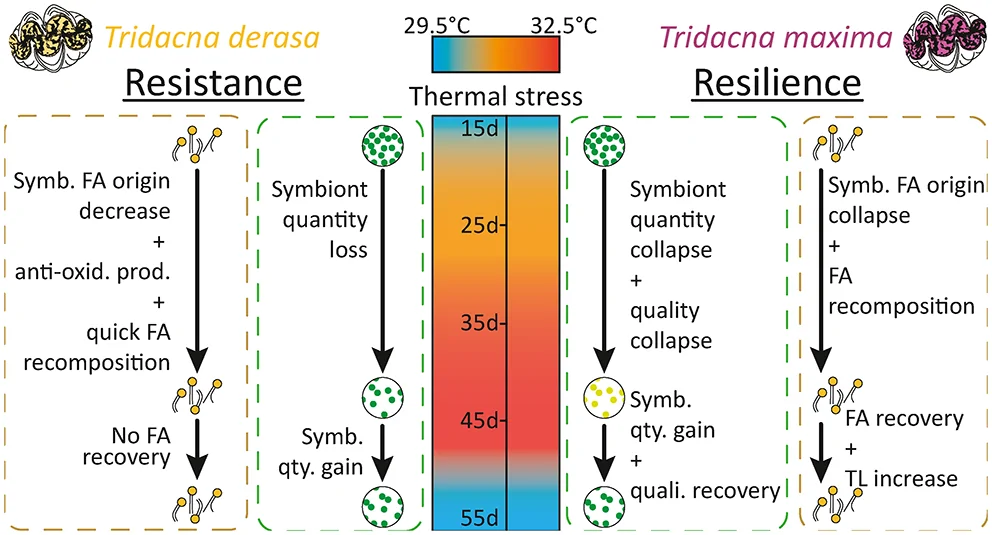

## 1 Introduction

Giant clams are ecologically and socio-economically significant inhabitants of the tropical Indo-Pacific region, serving as food source for humans and marine organisms, enhancing seabed topography and providing a shelter for numerous organisms such as gastropods, crustaceans, and sponges.^1–3^ They also play a crucial role in mitigating coastal eutrophication, and their cultural significance is recognized by many communities.^3,4^ Giant clams are symbiotic organisms, sharing nutrients and photosynthates with dinoflagellates from the *Symbiodiniaceae* family^5^ that reside extracellularly inside a tubular system in their mantle (Zooxanthellal Tubular System^6^). During optimal environmental conditions, *Symbiodiniaceae* can provide enough photosynthate to meet the daily energetic requirements of most giant clam species.^7–9^ In combination with their efficient filtration capacity,^9^ this source of autotrophic carbon provides giant clams with abundant food resources, allowing them to thrive in oligotrophic conditions.^10,11^

However, global warming, with sea surface temperatures across the Indo-Pacific region projected to rise by approximately 2–4 °C by 2100 under high-emission scenarios (SSP3-7.0),^12^ is expected to impact the autotrophic capacity of giant clams. Both high temperature and light intensity are factors known to drastically reduce the photoefficiency of *Symbiodiniaceae* and greatly increase the production of reactive oxygen species (ROS).^13,14^ Once the production of ROS exceeds the regulatory capacity of the antioxidant system, oxidative damage occurs,^15–17^ leading to symbiont apoptosis and bleaching.^18–20^ Consequently, during stress conditions, giant clams experience oxidative stress and a significant, if not complete, loss of their autotrophic food sources, thereby challenging their basal metabolisms.^18,21,22^

Metabolic processes are regulated by lipid availability, with lipid reserves being extensively catabolized to sustain energy requirements in adverse conditions. Therefore, during stress, total lipid concentration (TL) is commonly used to monitor the organism’s overall health status.^23–25^ In corals, fatty acids (FAs) are the major constituents of lipids, and FA composition has proven to be an efficient tool to study both nutrition and physiological response to environmental stress.^26–28^ The FA composition of the cellular membrane adapts to temperature changes to preserve optimal membrane fluidity and metabolic activity.^29–31^ Yet, temperature is only one factor that influences the composition of FAs, and other factors, such as food availability, must also be considered.^32,33^ The biosynthesis of FAs is metabolically demanding, and as a result, marine organisms largely derive them from their diet. Marine animals can produce saturated fatty acids (SFA, e.g., C16:0 and C18:0) and monounsaturated FAs (MUFA, e.g., C16:1n-7 and C18:1n-9) *de novo*. However, it is widely recognized that most marine animals cannot produce polyunsaturated fatty acids (PUFA) *de novo* due to their lack of Δ12 and Δ15 desaturases, which prevents the biosynthesis of C18:2n-6 and C18:3n-3,^34–36^ essential building blocks for the creation of other PUFAs, such as membrane components or anti-inflammatory precursors.^28,37,38^ In marine ecosystems, photosynthetic and microbial organisms are usually considered as primary sources of C18:2n-6 and C18:3n-3, and these essential FAs are, therefore, commonly used as diet tracers to evaluate trophic strategies and physiological responses under normal conditions or with environmental stress, such as thermal anomalies.^23,34,39,40^

The contribution of symbionts to giant clam nutrition differs from one species to another, suggesting that not all species of giant clams will be equally impacted by thermal stress.^10,41–43^ Additionally, giant clams host different genera of *Symbiodiniaceae*; *Symbiodinium*, *Cladocopium*, *Breviolum*, *Durusdinium,* and/or *Gerakladium*,^10,44–46^ which may further influence species-specific responses to environmental stressors. However, despite the understanding of the FA composition in giant clams^47^ and their global evolution after short thermal stress,^18^ existing literature fails to address the potential variability in response and recovery processes among different giant clam species following prolonged thermal stress. In this study, we conducted a long-term thermal stress experiment, followed by a brief recovery period on two giant clam species, *Tridacna maxima*, which is known for its moderate dependence on *Symbiodiniaceae*, and *Tridacna derasa*, which exhibits greater reliance on *Symbiodiniaceae*.^9,10,48^ The health status of the giant clams was monitored through visual color assessment, and the *Symbiodiniaceae* density and photosynthetic efficiency were measured. The TL and FA compositions of the two host species and their symbionts were also evaluated. This study aimed to understand (i) the impact of long-term thermal stress on each host and their respective symbiont population and (ii) if the difference in symbiont reliance also implies divergent lipidomic responses under stress conditions.

## 2 Materials and Methods

### 2.1 Experimental Design

One hundred twenty hatchery-cultured giant clams of two species, *T. maxima* (*n* = 60) and *T. derasa* (*n* = 60), of similar average sizes (9.5 cm and 8.3 cm, respectively), were collected at 2–5 m depth from a 30 m^2^ sandy area, at the Tabunan lagoon ocean nursery in Semirara Island (Philippine, 12°05′13.62″ N, 121°20′45.66″E) located near the Semirara Marine Hatchery Laboratory. A thermal stress experiment was performed in eight open-circuit flow aquariums (58 L) placed in a raceway. Each aquarium contained fifteen individuals of the same species (Supporting Information—Figure S2). All aquaria were supplied with seawater pumped directly from the adjacent lagoon, and a coarse filter was used upstream of the aquaria to minimize sediment load. The experiment was performed outdoors under a light-permeable transparent roof supplying equivalent light as natural conditions^49,50^ (Supporting Information—Figure S3). In each aquarium, the maximum quantum yield (*Fv/Fm*) of five giant clams was measured daily during the thermal stress, or every other day during the recovery, using a diving pulse amplitude-modulated fluorometer (DIVING-PAM Heinz Walz GmbH, Effeltrich, Germany), after dark adaptation at 04:00 in the morning or during daylight at noon.

Four control aquariums were maintained at average lagoon temperature (29.5 °C), while four others were exposed to thermal stress for five weeks (maximum of 32.5 °C, +3 °C, intermediate value within the projected end-of-century warming range, SSP3-7.0; IPCC, 2021). A chiller (Hailea HC-500A) was used to maintain the raceway temperature at 29.5 °C, while temperature controllers (Inkbird ITC-608T) and heaters (Aquael Easyheater) were used to increase the temperature in aquariums subjected to thermal stress. In all aquariums, a circulation pump was used to ensure the uniformity of seawater temperature. Water quality parameters, including salinity, pH, and dissolved oxygen (DO), were monitored regularly throughout the experiment in each aquarium. Across all tanks, salinity ranged from 30.2 to 33.0 ppt, pH from 8.05 to 8.60, and DO from 6.82 to 8.56 mg L^–1^. Temperature/Light Data Loggers (UA-002, Onset, Bourne, Massachusetts) were used to record the temperature and light data every 15 min in each aquarium.

After 15 days of acclimation, four aquaria (*n* = 2 aquaria per species) were subjected to thermal stress temperature (ST), while the other four (*n* = 2 aquaria per species) were maintained at lagoon temperature, hereafter referred to as control temperature (CT). To mimic natural thermal stress, a temperature ramping regime was carried out as follows: 0.5 °C day^–1^ increase for 2 days (30.5 °C by day 16), 5 days hold, 0.5 °C day^–1^ increase for 2 days (31.5 °C by day 22), 5 days hold, 0.5 °C day^–1^ increase for 2 days (32.5 °C by day 28), and 18 days hold. After thermal stress, the temperature was returned within 36 h to 29.5 °C and maintained for 7 days to observe recovery (Supporting Information—Figure S4). Samples were collected on day 15 (T0; CT = 29.5 °C), day 27 (T1 = short ST; CT = 29.5 °C and ST = 31.5 °C), day 46 (T2 = long ST; CT = 29.5 °C and ST = 32.5 °C), and day 54 (T3 = recovery; CT and ST = 29.5 °C). Clam sampling was performed in the mornings, and temperature adjustments were performed in the evenings. For each species, the mantle of six giant clams was sampled per time and condition (*n* = 42 total samples per species), immediately placed on ice, and stored at −20 °C within an hour prior to further analysis. Given the substantial amount of mantle tissue required for stable isotope analyses, only unsampled individuals were released into the lagoon for recovery. All sampling and export of giant clams were conducted under the Department of Agriculture Bureau of Fisheries and Aquatic Resources Gratuitous Permit 0169-19 and CITES Export Permit 4946.

### 2.2 Sample Preparation

#### 2.2.1 Host and Symbiont Separation

Mantle processing served two objectives: (i) separating a pure symbiont and a pure host fraction for independent lipid analysis and (ii) preparing aliquots to estimate symbiont density. Mantle samples were weighed, and a single 1.5 g piece was used for both lipid extraction and symbiont counting. To separate symbionts from the host tissues, the mantle was lacerated with a razor blade, placed in a 50 mL centrifuge conical tube filled with 6 mL of Milli-Q water with butylated hydroxytoluene (BHT) solution to prevent lipid oxidation, and then sonicated (S-150D, BRANSON, United-States) for 45 s at 5 W to leak the symbionts out of the giant clam’s zooxanthellal tubular system. This 6 mL symbiont extraction was saved, and another round of sonication using fresh Milli-Q water was performed on the same lacerated mantle to maximize symbiont extraction. The 50 mL tube with the lacerated mantle was set aside in ice for further processing. The two 6 mL extractions were pooled together (12 mL), and an aliquot of 500 μL was saved for symbiont counting. The remaining symbiont fraction (11.5 mL) was further processed to obtain a pure fraction of symbiont before lipid analyses. Symbionts were pelleted by centrifugation for 10 min at 3000 RCF and 4 °C, and the supernatant was discarded. Three more cycles of dilution in 5 mL of Milli-Q water with BHT, followed by centrifugation for 10 min at 3000 RCF and 4 °C, were performed to remove residual salt and particles of host tissues to obtain purified symbiont pellets. The mantle previously lacerated and set aside was then homogenized with a Tissue-Tearor (Ultra Turrax T25, IKA labortechnik staufen, Germany) in 7 mL of Milli-Q water with BHT. A new aliquot of 500 μL was saved for symbiont counting to account for the symbionts not extracted during the first steps of the protocol. The remaining homogenate (6.5 mL) was used for lipid analysis and centrifuged at 2000 RCF for 10 min and 4 °C to separate giant clam host tissue from any residual symbionts. The supernatant containing host tissues was collected and centrifuged again for 5 min at 2200 RCF and 4 °C to obtain a purified host fraction. Both host and symbiont purified fractions were stored at −40 °C overnight and then freeze-dried for lipid analyses (Alpha 1–4 LCS-Plus, Christ, Germany).

#### 2.2.2 Symbiont Counting

The two 500 μL symbiont aliquots saved during the host and symbiont separation were processed independently. Before counting, two drops of Lugol’s iodine solution were added to each aliquot. Samples were transferred to a hemocytometer with a glass pipette and examined under an optical microscope (objective 40×, Olympus CHK) with a magnification of 400×. Symbionts located in the four 1 mm^2^ corners of the hemocytometer were counted. Accuracy and consistency were set by maintaining the symbiont concentration in the range of 100 cells mm^–2^, using dilution with mineral water when needed. The procedure was repeated 3 times minimum and was stopped when the standard deviation of the counts was less than 10%. The symbiont density was standardized by the wet mantle mass. Finally, the symbiont density of each organism was obtained by summing the concentration of the two symbiont aliquots.

#### 2.2.3 Lipid Extraction

Following freeze-drying, a predetermined weight of pure dry host tissue (10 ± 0.2 mg) and pure dry symbionts (5 ± 0.2 mg) was homogenized with the Tissue-Tearor in 30 mL of ice-cold Folch solution (2:1; methanol/chloroform v/v) to perform TL and subsequent targeted FA extraction. An aqueous sodium chloride solution (0.9%; w/v) was used to separate lipids from polar molecules. Nonadecanoic acid (19:0; 1 μg L^–1^) was added as an internal standard. Total lipids were quantified after drying the sample under a nitrogen gas stream. Lipids were resuspended in hexane and FAs transformed into FA methyl esters (FAMEs) with a sulfuric acid/methanol solution (1:40; v/v) at 100 °C for 1 h. The reaction was stopped with deionized water, and FAMEs in hexane were separated from deionized water after 5 min centrifugation at 2500 RCF and 4 °C. The extracted FAMEs were placed on a heating block at 37 °C, dried under nitrogen gas, and then resuspended in 150 μL of hexane for gas chromatography–mass spectrometry (GC–MS) analysis set to electron ionization. Only 1 μL of the derivatized sample was inserted in the GC column, using a split ratio of 100:1. A 1 mL min^–1^ flow of helium gas carried the samples through the GS-MS system (Agilent 5977A mass selective detector interfaced with an Agilent 7890B gas chromatograph) via an SP-2560 capillary column (100 m × 0.25 mm, 0.2 μm film thickness, Sigma-Aldrich, USA). Elution of the sample was done by gradually increasing the temperature from 100 °C to 200 °C by 4 °C min^–1^, 200 °C to 220 °C by 1 °C min^–1^, and from 220 °C to 240 °C by 5 °C min^–1^. Identification of 21 FAMEs was performed using an external standard (37 Component FAME mix, Supleco, USA) and specific retention time. Each FAME peak was quantified by calculating the proportion of its integral (i.e., peak area) to that of the internal standard. FAME concentrations (%) were normalized to total fatty acids (TFAs).

### 2.3 Statistical Analyses

Normality, homoscedasticity, and homogeneity between sampling times were assessed using Shapiro–Wilk, Levene, and Kruskall–Wallis tests to ensure the selection of appropriate statistical analyses. Additional density plots and median absolute deviation computations facilitated the identification and subsequent removal of five and six outlier samples from the symbiont density and lipid data analyses, respectively. Additionally, a few samples could not be used in all statistical tests due to missing values in specific variables and were removed when needed (total missing value, *n* = 12). The absence of a batch effect was confirmed by examining box plots and performing Kruskal–Wallis tests. Species and tissue differences between control samples were assessed with Wilcoxon tests. Statistical effects of the temperature on lipid concentration were assessed using the Kruskal–Wallis test on ratios of each stressed organism’s value to the average value of unstressed organisms sampled at the same time (hereafter named “average analogue control”) to remove potential experimental bias. Bleaching was tested using the symbiont ratio density over average analogue control with one-way ANOVA. Differences between time conditions were tested with the paired-samples Wilcoxon post hoc test adjusted for Bonferroni correction or Tukey’s HSD post hoc tests when one-way ANOVA was performed. Principal canonical analyses (PCAs) were used to highlight the predominance of the FA concentration in the host or symbiont for both species studied. To account for low-symmetry distributions, the 21 FAs were square-root-transformed prior to this analysis.^51^ The TL values were standardized using the sample dry weight, while TFA was standardized using TL prior to analyses.

Finally, to test the effects of heat stress and recovery on the symbiont photosynthetic efficiency, *Fv/Fm* time series were decomposed into three periods: short and moderate temperature increase (T0–T1), long and severe temperature increase (T1–T2), and recovery treatment (T2–T3). Best-fit models for each species, condition, and time period were selected by comparing linear, log-linear, and quadratic models using Akaike’s Information Criterion (AIC). Model slopes (percentage change per day) were then compared between conditions (i.e., control and stress conditions) using two-tailed t tests on estimated marginal trends.

All tests were performed on R version 4.2.3 (R Core team, 2023), and results were considered significantly different for *p*-values ≤ 0.05. In addition to the base package embedded in R, the following packages were used: “readr”^52^ and “readxl”^53^ for importing and exporting dataframes; “dplyr”,^54^ “Rmisc”,^55^ “stringr”,^56^ and “tidyr”^57^ for data management; “car”,^58^ “ggpmisc”,^59^ “lsmeans”,^60^ “emmeans”,^61^ and “vegan”^62^ for statistical analyses; and “ggplot2”,^63^ “ggthemes”^64^ and “multcompView”^65^ for graphical purposes.

## 3 Results

### 3.1 Symbiont Density and Photosynthetic Efficiency

Under control conditions, the symbiont density of *T. derasa* was significantly higher compared to that of *T. maxima* (39.3 ± 11.6 ×10^6^ g^–1^ and 8.3 ± 5.0 × 10^6^ g^–1^, respectively; Wilcoxon signed-rank test *p* val < 0.05). Thermal stress significantly impacted the two species, both reaching a minimum of symbionts at T2 with a loss of 55.92 ± 19.4% of symbionts for *T. derasa* and 89.08 ± 6.23% for *T. maxima* (Figure 1, Supporting Information—S5 ANOVA and post hoc test). This greater loss of symbionts at T2 in *T. maxima* (Figure 1; pairwise Wilcoxon test: *p* < 0.05) matched the pronounced bleaching of *T. maxima*, in contrast to the paling of *T. derasa* (Figure 2). While not statistically significant, a divergent dynamic in the recovery of symbiont density is visible between the two giant clams, with *T. maxima* displaying a faster and more pronounced increase in symbionts at T3 (Figure 1). The photosynthetic efficiency of the remaining symbionts greatly differed between the two hosts. Based on AIC, log-linear models were selected to compare the estimated marginal trends’ slope variation between conditions (Supporting Information—Table S5). During T0–T1, no treatment differences were detected: *T. derasa* (control = + 0.58% day^–1^, stressed = + 0.58% day^–1^, *p* = 0.978) and *T. maxima* (control = + 0.58% day^–1^, stressed = + 0.30% day^–1^, *p* = 0.207). During T1–T2, stressed *T. maxima* showed a strong decline (−1.50% day^–1^) compared to controls (+0.13% day^–1^, *p* < 0.001), while *T. derasa* showed no treatment difference (control = −0.15% day^–1^, stressed = −0.25% day^–1^, *p* = 0.314). Finally, during T2–T3, stressed *T. maxima* increased rapidly (+5.87% day^–1^) versus no change in controls (−0.26% day^–1^, *p* < 0.001), whereas *T. derasa* showed no treatment difference (control = + 0.30% day^–1^, stressed = −0.63% day^–1^, *p* = 0.298).

**Figure 1:**
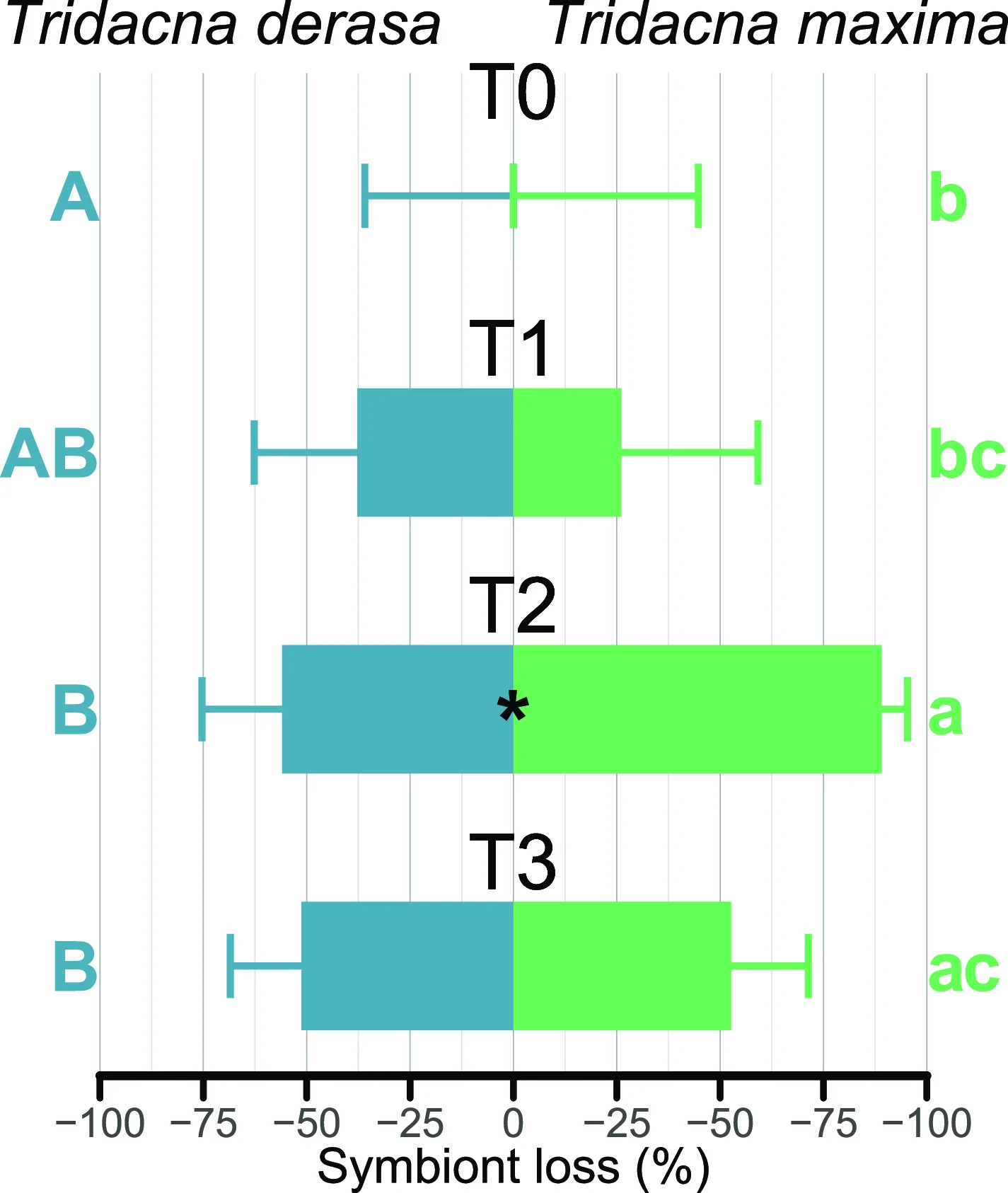
Relative symbiont density losses of *Tridacna derasa* (blue) and *Tridacna maxima* (green). Independent one-way ANOVA were used for each giant clam (ANOVA test: *p*.val < 0.05 for both species). Letters indicate statistical differences between times (Tukey’s HSD ANOVA test: *p*.val < 0.05). * denotes significant differences between the two species (pairwise Wilcoxon test: *p*.val < 0.05).

**Figure 2:**
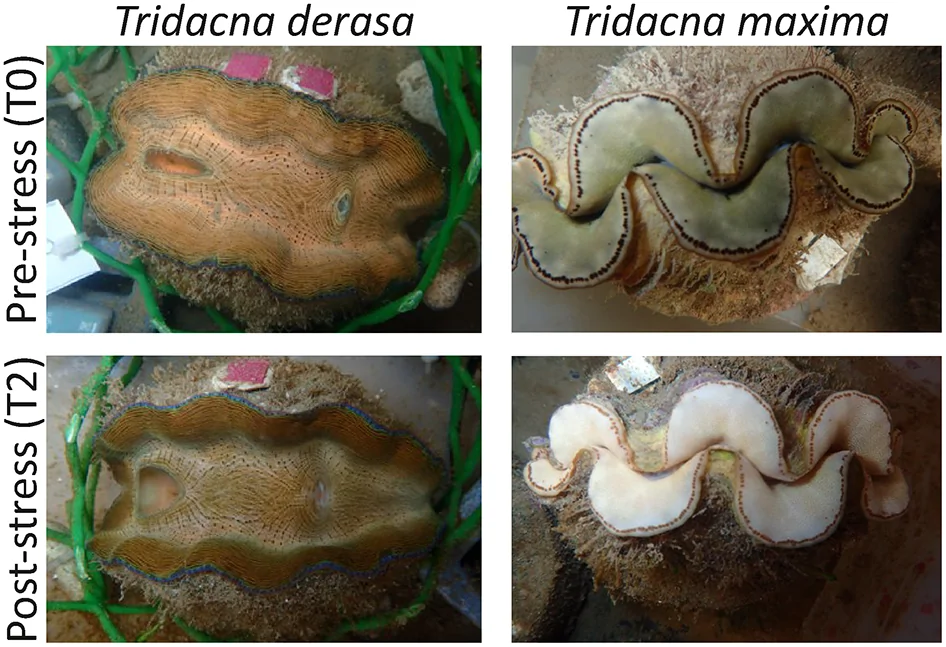
Example of the visual differences between *Tridacna derasa* and *Tridacna maxima* before (T0) and after heat (T2) stress.

### 3.2 Lipid Concentration and FA Composition

#### 3.2.1 Total Lipids and Total FA

Under control conditions, the proportions of TL relative to dry weight were similar among *T. maxima* host tissues, *T. maxima* symbionts, and *T. derasa* host tissues, being 34.26 ± 4.52%, 34.79 ± 6.67%, and 39.22 ± 10.49%, respectively (Supporting Information—Table S6). However, the TL percentage of *T. derasa* symbionts was nearly double, reaching 61.98 ± 18.19%. These proportions were not significantly impacted by thermal stress (Kruskal–Wallis test and Wilcoxon post hoc test, Supporting Information—S3, Kruskal–Wallis and post hoc test). Interestingly, after eight days of recovery from bleaching, the TL proportion of *T. maxima* host tissues significantly increased by 78.23 ± 27.06% compared to its average analogue control (Supporting Information—Table S7). The proportion of TFA within TL was higher in the host tissues of the two species (*T. derasa*: 18.20 ± 5.12%; *T. maxima*: 23.55 ± 6.01%) compared to their respective symbionts (*T. derasa*: 14.09 ± 4.81%; *T. maxima*: 17.39 ± 4.40%). However, TFA remained constant through thermal stress in the host and symbionts of both species. The increased TL concentration in *T. maxima* host tissues led to a significant decrease in TFA proportion. Due to this discrepancy, the following FA analyses are reported as relative proportions of TFA.

#### 3.2.2 FA Composition

##### 3.2.2.1 Control Condition

Overall, FA composition in the host and symbionts of both species was similar and mainly composed of SFAs (43–53%), with a majority of C16:0 (28–34%) and C18:0 (11–14%). PUFAs (36–43%) also accounted for a significant proportion of TFA, while MUFAs were only reported to a minor extent (8–18%) (Supporting Information—Tables S8–S10).

Species-specific PCAs with control samples from all sampling times highlighted an uneven distribution of some FAs between symbionts and host tissues (Figure 4). The first principal components of the PCAs explained 63.28% and 39.02% of the total variation for *T. derasa* and *T. maxima*, respectively, showing important differences between the host and symbiont FA composition. Most of these differences were driven by PUFA concentrations, with shorter chains found in greater proportion in symbionts (e.g., C18:3n-6 or C18:4n-3) and longer chains predominant in host tissues (e.g., C20:4n-6 and C22:4n-6). MUFAs were also mainly correlated with host tissues (e.g., C18:1n-9 and C20:1n-9). The second principal component accounted for the variability within the control samples of *T. derasa* (19.56%) and *T. maxima* (21.63%). Consequently, the anticorrelated C18:3n-6 and C18:3n-3 PUFAs were highly variable in the symbionts and the host tissues, respectively. In addition to host and symbiont FA composition differences, important variations between species were also observed. For example, C18:4n-3 proportions were three times higher in *T. derasa* symbionts (17.53 ± 4.10%) than that in *T. maxima* (5.89 ± 3.51%), while the proportion in host tissues remained similar for both species (*T. derasa*: 3.58 ± 0.64%; *T. maxima*: 4.73 ± 1.27%). Other important differences include the negligible proportions of C18:3n-6 in *T. derasa* tissues (host: 0.71 ± 0.45%; symbiont: 1.99 ± 0.86%) compared to *T. maxima* (host: 5.20 ± 1.90%; symbiont: 6.65 ± 3.00%). In contrast, C18:1n-9 was twice as abundant in *T. derasa* (host: 11.87 ± 1.30%; symbiont: 5.57 ± 2.20%) as in *T. maxima* (host: 5.11 ± 0.66%; symbiont: 2.70 ± 0.65%). Similar differences in C18:3n-3 proportions were also observed.

**Figure 3:**
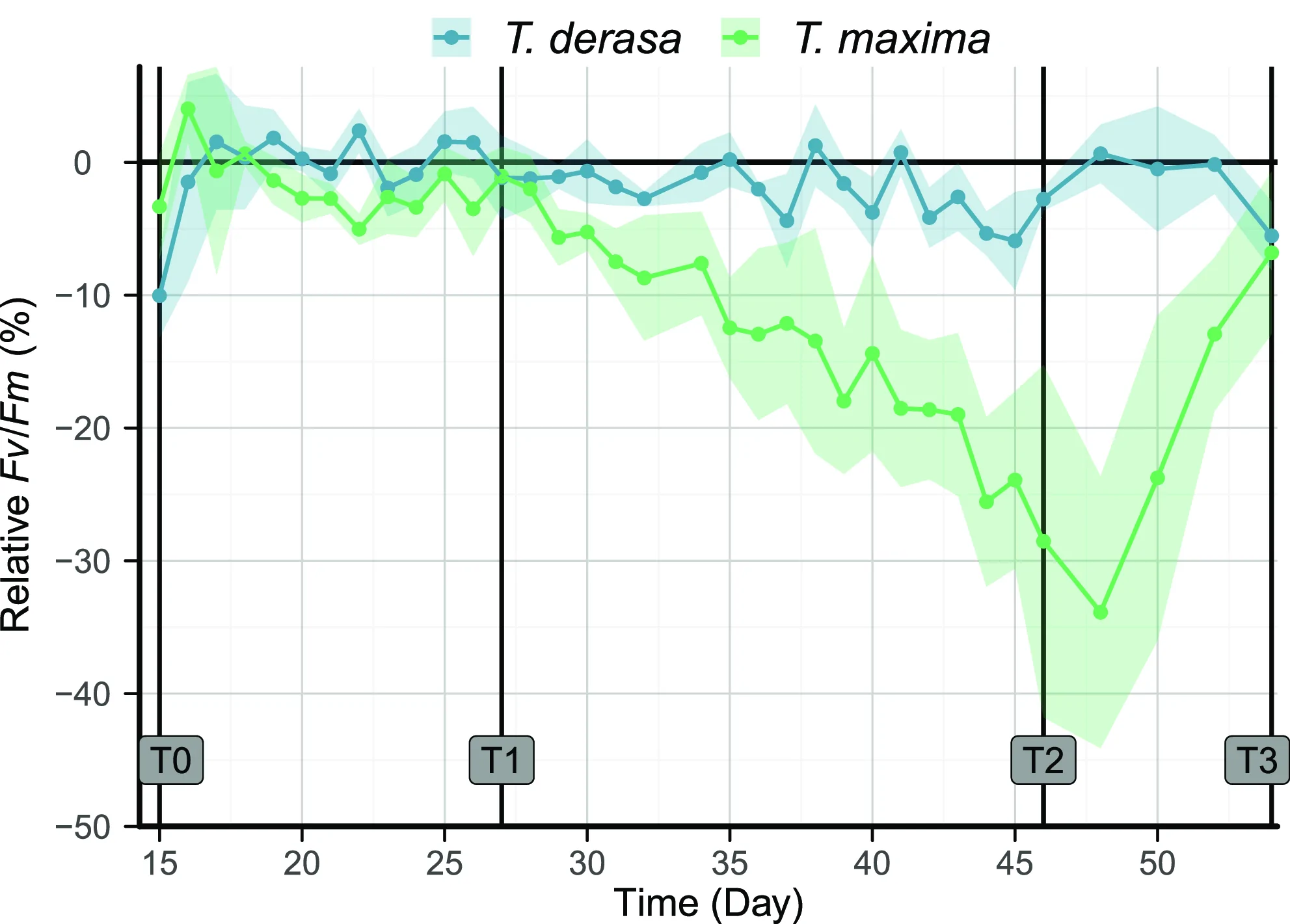
Relative *Fv/Fm* evolution of *Tridacna derasa* (blue) and *Tridacna maxima* (green) during the experiment. Solid lines and dots represent the average ratio of the stressed organisms over their average analogue control (*n* = 5). Shaded bands indicate the standard deviation of the same ratio. Significant differences between stress and control samples were tested for the periods T0–T1, T1–T2, and T2–T3. No significant change was observed for *Tridacna derasa* in any period. Conversely, the results for *Tridacna maxima* were highly variable, with the *Fv/Fm* values of the stressed organisms becoming progressively lower than their control values during T1–T2 and then catching up significantly during T2–T3.

**Figure 4:**
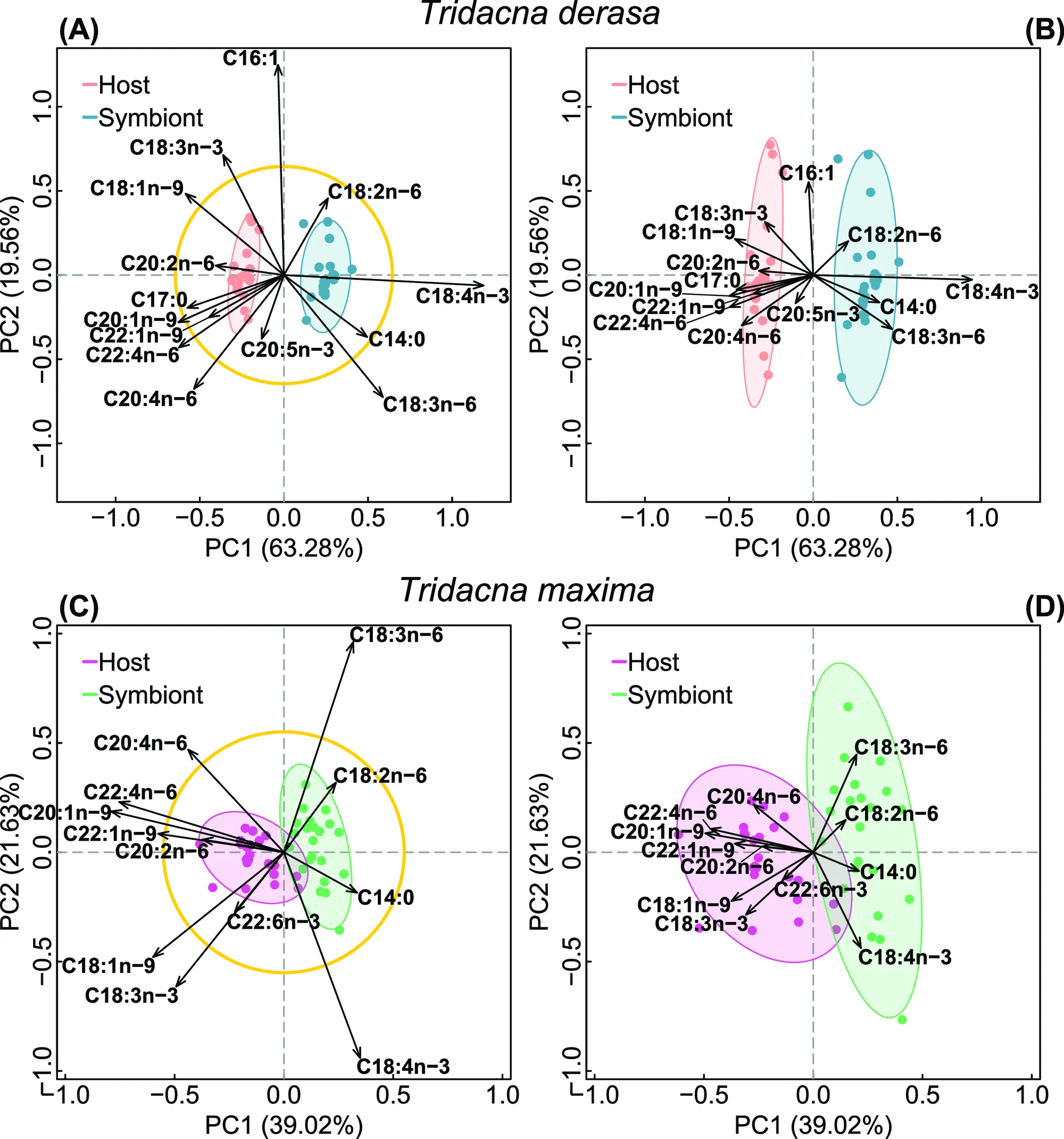
PCAs of the FAs from control samples of *Tridacna derasa* (top) and *Tridacna maxima* (bottom). Dots and ellipses (95% confidence interval) stand for hosts and symbiont replicas in control conditions. The circle of equilibrium (yellow) is represented on the scaling 1 biplot (Panels (A,C); accurate distances). For clarity, only lipids with a radius greater than 60% of the circle of equilibrium are represented. Panels (B,D) stand for the scaling 2 biplot (accurate angles).

##### 3.2.2.2 Thermal Stress Conditions

Although the thermal stress had only a minor effect on TL and TFA concentrations, the composition of FA classes changed substantially in both species throughout the experiment (Figure 5 and Supporting Information—Tables S8–S10). While the proportion of SFA and MUFA did not show statistically significant variation over time, the proportion of SFA globally increased in both the host and symbiont samples (Figure 5 and Supporting Information–Tables S8—S10). Conversely, PUFA proportions decreased significantly (Figure 5 and Supporting Information–Tables S8–S10). Although both species exhibited similar patterns of FA class changes under thermal stress, species-specific differences remained apparent with a maximum decrease of PUFA occurring at T1 for *T. derasa* (host: −10.41 ± 2.64%; symbiont: −18.89 ± 9.91%) and at T2 for *T. maxima* (host: −10.18 ± 2.41%; symbiont: −27.29 ± 12.36%). These interspecies differences were highly consistent in all FAs (Figure 5 and Supporting Information—Tables S8–S10). These combined changes resulted in elevated SFA/PUFA ratios in both species, with higher ratios in symbiont than host tissues (Figure 5 and Supporting Information—Tables S8–S10). Once again, the most substantial changes were observed at T1 for *T. derasa* (host: 21.91 ± 6.78%; symbiont: 44.76 ± 28.16%) and at T2 for *T. maxima* (host: 18.3 ± 6.42%; symbiont: 67.61 ± 49.44%). Further analysis of specific FA variations underlined important changes in the FA composition of the two species (Supporting Information—S3 Kruskal–Wallis and post hoc test). Throughout the experimentation, PUFA C18:2n-6 decreased in both species, reaching a minimum at T3. Significant decreases in the proportion of short unsaturated fatty acid (UFA) chains, such as C18:1n-9 (T2 = −10.59 ± 3.57%) and C18:3n-3 (T1 = −92.99 ± 1.76%), were also observed in *T. derasa* host tissues. In *T. maxima*, important decreases were observed for C18:3n-6 (T3-host = −62.9 ± 8.75%, *p* val = 0.056; T2-symbionts = −68.11 ± 12.62%, *p* val < 0.05) and C18:4n-3 (T3-host = −45.44 ± 15.81%, *p* val < 0.05; T2-symbionts = −74.21 ± 17.8%, *p* val < 0.05). In contrast, the proportions of several predominant long UFA chains increased significantly. In *T. maxima*, the proportion of C20:1n-9 (T2-host = +32.35 ± 10.69%; T2-symbionts = +88.78 ± 19.05%), C20:4n-6 (T3-host = +30.44 ± 14.58%; T2-symbionts = +54.56 ± 39%), and C22:4n-6 (T3-host = +37.25 ± 23.05%; T2-symbionts = +66.37 ± 21.06%) significantly increased. Comparatively, these FAs also increased in *T. derasa* hosts and symbionts, although only C20:1n-9 reached statistical significance (T2-host = 29.5 ± 11.46%). During thermal stress, C20:5n-3 was the only long UFA to show a significant decrease in the host tissues of both species (*T. derasa* at T2 = −17.91 ± 4.04%; *T. maxima* at T2 = −19.76 ± 3.88%) and a pronounced decrease in *T. derasa* symbionts (T1 = −53.04 ± 10.96%).

**Figure 5:**
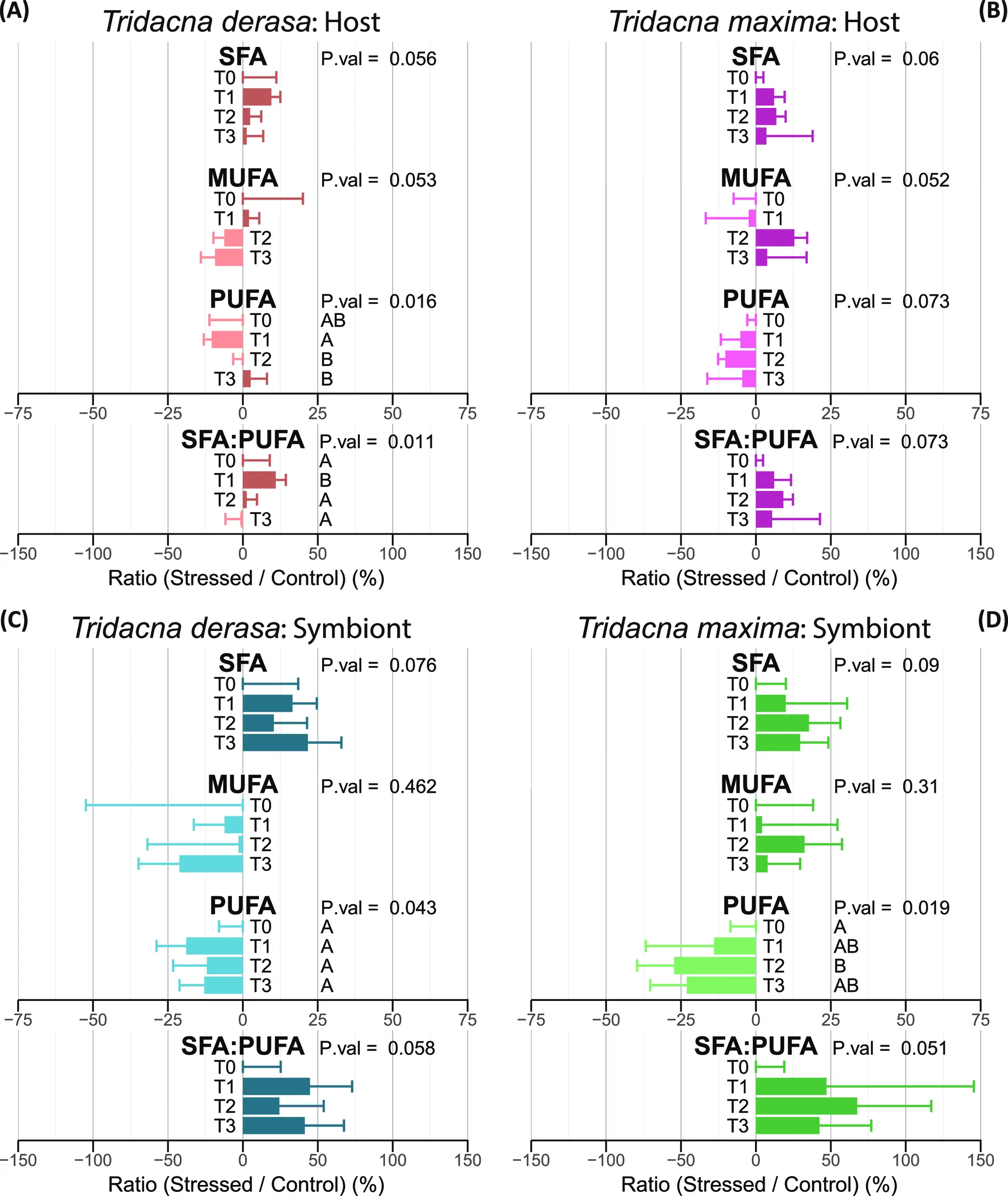
Relative FA classes of both host (A,B) and symbionts (C,D) of *Tridacna derasa* (A,C) and *Tridacna maxima* (B,D). The average and standard error of each FA class in stressed conditions over their average analogue control is presented for each sampling time. Statistical tests were carried out to detect differences in each FA class concentration over time. *P*.vals stand for Kruskal–Wallis results performed on each FA class. When significant (*p*.val < 0.05), post-hoc pairwise Wilcoxon tests with Bonferroni corrections were performed to detect which times differ from the others. Combinations of letters have been assigned to times to differentiate significant differences (*p*.val < 0.05).

## 4 Discussion

Bleaching is a common threat to marine symbiotic species and has been documented in giant clams, notably during mass bleaching events.^46,66^ Despite their high filtration capacity, giant clams rely on their symbionts to cover their energy demand,^8,67^ making them susceptible to thermal stress from bleaching-induced symbiont loss, which in some cases can lead to mortality.^68,69^ However, interspecies physiological responses to thermal stress remain poorly documented. We compared the thermal stress responses and recovery of two giant clam species of similar sizes from the same lagoon environment by integrating lipidomic analysis with the measurements of *Symbiodiniaceae* density and photosynthetic efficiency, revealing distinct coping strategies in each species. *T. maxima* exhibited intense bleaching but rapid recovery of symbiont density and a significant increase in TL during the recovery, indicating a resilience strategy characterized by rapid metabolic adaptation. In contrast, *T. derasa* exhibited relatively stable symbiont density and TL levels throughout, reflecting a resistance strategy based on greater symbiont reliance. Alteration in FA composition occurred at different rates and intensities between species, further underscoring the species-specific responses to thermal stress. This work advances our understanding of the mechanisms underlying responses to thermal stress, providing critical insights into predicting species vulnerability and guiding targeted conservation efforts in the face of climate change.

### 4.1 Baseline and Stress-Induced Variation in Symbiont and Lipids

Despite being mixotrophs, giant clams’ trophic niches differ between species.^10^ *T. derasa* mostly relies on its symbiont to meet its respiratory needs,^9^ while *T. maxima* symbionts contribute to only half of their metabolic carbon requirement.^48^ In this study, *T. derasa* hosted four times more symbionts than *T. maxima* under control conditions, indicating a different symbiont density baseline between species and confirming a greater reliance on autotrophic resources in *T. derasa*.

The thermal stress impacted both species differently, with changes in symbiont density and total lipid content. At elevated temperatures, *T. maxima* bleached (Figure 2), experiencing a high decrease in both symbiont density (89%) and photosynthetic efficiency, likely resulting in a negligible autotrophic supply (Figures 1 and 3). In contrast, *T. derasa* only paled, with the photosynthetic efficiency remaining unaffected, suggesting the maintenance of a substantial autotrophic supply despite a 50% loss in symbiont density. These results suggest a greater thermal resistance in *T. derasa*, which may be attributed to different symbiont assemblages.^10,44,46^ Indeed, symbiont community analysis (Supporting Information—Table S11) revealed that *T. derasa* was predominantly associated with *Cladocopium* (92.3%, mostly C93a-C93e-C93n-C93ac profiles), whereas *T. maxima* was dominated by *Symbiodinium tridacnidorum* (95.9%, mostly A3-A3cj profiles). *Symbiodiniaceae* diversity covers a large spectrum of temperature and light intensity preferences, which can influence the robustness of the symbiosis and species resilience,^46^ although the thermal tolerance of specific phylotypes associated with giant clams remains poorly characterized. Despite having an autotrophic supply advantage under stress, the TL of *T. derasa* remained similar to that of *T. maxima*. This discrepancy indicates that *T. derasa* is more reliant on its symbionts to sustain energy reserves under stress.

Recovery responses also differed between species. Unlike *T. derasa*, which maintained a low symbiont density yet stable photosynthetic efficiency, *T. maxima* demonstrated rapid recovery in both respects. Symbiont recovery coincided with a 78% increase in TL in host tissues, which was not reflected in its FA concentration (Supporting Information—Table S7). Higher TL may result from increased wax esters and sterols. Wax esters serve as energy reserves used during prolonged thermal stress,^34,70,71^ while sterols play a key role in the reorganization of the lipidic membrane.^18,72,73^ *T. maxima* tolerance of extreme symbiont loss is possibly related to its lipid class composition, and further investigation is required to explore other lipid classes to fully understand their functions in thermal stress resilience.

### 4.2 Effect of Symbiont Loss on FA Composition

Variations in symbiont density and photosynthetic efficiency co-occurred with variation in FA composition of the two species, highlighting (i) the importance of essential FA transfer between host and symbionts and (ii) the varied symbiont reliance in giant clam trophic strategy. A decrease in the FA C18:2n-6 was observed at the same time as the decline in symbiont density in both species, indicating a symbiont-derived origin, as also suggested for other marine holobionts.^34,74^ . In marine ecosystems, C18:3n-6 and C18:4n-3 are directly derived from shorter FAs that are not produced *de novo* by animals and are commonly identified as algal biomarkers.^28,35^ The proportions of these FAs differed in *T. derasa* and *T. maxima*, with only *T. maxima* showing a significant decrease in both under thermal stress (Supporting Information–Tables S8–S10, Supporting Information—S3 Kruskal–Wallis and post hoc test). The reduction of these FAs within host tissues can only be attributed to the severe bleaching experienced by *T. maxima* and the reduced FA proportions of its symbionts, ultimately limiting the transfer of C18:3n-6, C18:4n-3, or their precursors. Their proportions remained stable until recovery, subsequently increasing, mirroring the increase in symbiont density and photosynthetic efficiency. In contrast, proportions of C18:3n-6 and C18:4n-3 in *T. derasa* decreased only slightly, likely due to its higher symbiotic density and photosynthetic efficiency during thermal stress. However, these declines persisted even when favorable conditions returned, likely due to slower symbiont recovery in this species. Observed changes in host C18:3n-6 and C18:4n-3 concentrations alongside symbiont density and photosynthetic efficiency indicate that giant clams, like soft and hard corals, mainly rely on their symbionts to acquire essential short FAs.^34,35,74^

### 4.3 FA Recomposition in Response to Thermal Stress

Similar FA adaptations were observed in *T. derasa* and *T. maxima* under thermal stress conditions. An increase of SFA/PUFA ratio was observed in both hosts (Figure 5). This adaptation, widely reported in marine organisms,^34,75,76^ is usually attributed to membrane cell fluidity regulation^30^ or as a response to mitigate ROS damage; both adaptations ultimately limit cellular apoptosis.^15,17^ Although not mutually exclusive, the increase in the SFA/PUFA ratio in both species most probably reflects an adaptation to alleviate ROS damage, which correlates with the observed loss of symbionts and decrease in photosynthetic efficiency.^13,77,78^ SFA/PUFA ratios in *T. derasa* returned to normal levels during the extended stress period (T2), suggesting high production of antioxidant molecules to maintain symbiosis, substantial reduction in ROS production from symbionts, or a combination of both responses. In contrast, the SFA/PUFA ratio in *T. maxima* remained high until recovery. This suggests that the elevated expression of ROS scavengers, as previously observed in *T. maxima* by Dubousquet et al. in 2016,^18^ during thermal stress, may be insufficient to fully prevent oxidative damage (Figure 5). These results also highlight the importance of oxidative stress in giant clams and, therefore, the need to quantify it for future studies.

In addition, both species displayed an overall shift from short to longer FAs. Long-chain FAs have higher melting temperatures, which are thought to help endure heat stress.^30,79^ This explains the relative FA chain elongations in organisms living in warm environments.^18,80^ The drop of C18:3n-6 concentration in *T. maxima* host and symbiont tissues could also be explained by its translation into longer FAs, such as C20:4n-6 and C22:4n-6.^81^ This transformation involves elongases in addition to Δ6 and Δ5 desaturases, enzymes that have not yet been explored in giant clams, but have been described in many other marine organisms, including bivalves.^81^ A similar chain elongation could also explain the reciprocal patterns of C18:1n-9 and C20:1n-9 in *T. derasa*, with the latter being produced from the former.

### 4.4 *T. derasa’s* Unique Response to Cope with Inflammatory Stress

*T. derasa* exhibited a singular response to short thermal stress (T1) not observed in *T. maxima*. Soon after the temperature increase, the C18:3n-3 concentration in *T. derasa* host tissue dropped by 92%. This essential FA cannot be synthesized *de novo* by marine animals and must be acquired through filter feeding or lipid transfer from symbionts.^34,74^ Depletion of this lipid in *T. derasa* host tissue occurred before significant symbiont loss and despite stable levels in the symbionts (Supporting Information—Tables S8–S10). This reduction in host tissue may therefore result from the decreased transfer of this lipid from symbionts^39,82^ and/or increased utilization by the host. Alongside the drop in C18:3n-3, C20:5n-3 also decreased significantly in both the host and symbiont. As an anti-inflammatory precursor synthesized from C18:3n-3, C20:5n-3 availability has proved to be vital in many organisms during stress events to preserve viable metabolism.^37,76,83^ The decrease in C20:5n-3 was three times lower in the host than in the symbiont tissues. Therefore, this pattern suggests the conversion of C18:3n-3 into C20:5n-3 to compensate for the reduced lipid transfer from symbionts during thermal stress and/or the utilization of this FA by the host. Altogether, these results indicate the activity of elongases in addition to Δ6 and Δ5 desaturases in giant clams. Finally, the full restoration of C20:5n-3 proportion in both the host and symbionts during the recovery period also supports its critical role in the anti-inflammatory system of *T. derasa*.

### 4.5 Strategies and Ecological Implications

Under high-emission scenarios (SSP3-7.0), sea surface temperatures across the tropical Indo-Pacific region are projected to rise by 3 °C by 2100,^12^ with marine heatwaves increasing in frequency, intensity, and duration. These climate-driven stressors further threaten giant clams, by adding to existing pressures from overfishing and habitat degradation. Through the analysis of both symbionts and host tissues, we show that thermal stress simultaneously reduces symbiont density and depletes essential FAs that serve as precursors for hormonal and membrane functions. However, our work also demonstrates that the physiological implications of bleaching differ from one species to another. While *T. derasa* lost 56% of its symbionts, it maintained photosynthetic efficiency and rapidly adjusted its FA composition (e.g., SFA/PUFA, C20:5n-3), reflecting a resistance strategy. In contrast, *T. maxima* underwent dramatic bleaching (i.e., lost 89% of its symbionts) but exhibited rapid recovery of its symbionts and FA composition and even increased its total lipids by 78% compared to control condition within eight days, demonstrating a resilience strategy. We emphasize here that species-specific coping strategies cannot be reduced to a single “bleaching threshold” and that monitoring programs should not only track species-specific recovery rates and mortality but also species strategies to withstand thermal stress.

Beyond their ecological value, giant clams are important socio-economic organisms for many communities in the Indo-Pacific region.^4^ The contrasting strategies we demonstrated have direct implications for aquaculture and conservation. Understanding these species-specific coping mechanisms can help tailor management, with aquaculture operations adjusting depth, shade, or stock relocation to each species’ response.^69^ Understanding whether resistance (*T. derasa*) or resilience (*T. maxima*) confers a long-term advantage under repeated heatwaves is a priority for predicting future reef communities and managing these socio-economically valuable species.

## Supplementary Material





## Data Availability

All scripts used for all analyses are available via the Zenodo repository https://doi.org/10.5281/zenodo.19822377.

## Notes

Raw data and statistical results (XLSX)
Schematic representations and graphics of the experimental setup, light intensities, aquarium temperatures, detailed data frames of symbiont photosynthetic efficiency results, variation of all FAs studied, and symbiont clade percentages (PDF)
