# Supplementary material for: Divergent Giant Clam Strategies to Cope with Thermal Stress Revealed by Fatty Acid Dynamics
Source: Environ Sci Technol. 2026 Jul 2;60(33):23245–55. doi: 10.1021/acs.est.6c03210 (PMC13523697; doi:10.1021/acs.est.6c03210)
Supplement: Supplementary file 1 [file es6c03210_si_002.pdf]

Supplementary material

Title: Divergent giant clam strategies to cope with thermal stress revealed by fatty acid dynamics
Running title: Giant clams' species-specific response to thermal stress

Isis Guibert[1-2]*, Leonard Pons[1-3]*, Taihun Kim[3], Jane Ching Yan Wong[1], Ava Yee Tung Chan[1], Patrick Cabaitan[4], Cecilia Conaco[4], Jetty Chung-Yung Lee[1], David Michael Baker[1]†.

*Co-first author
†Corresponding authors: David M. Baker

Affiliations

1 - Swire Institute of Marine Science, The University of Hong Kong, Hong Kong, SAR, China.
2 - School of Life Sciences, Simon F.S. Li Marine Science Laboratory, The Chinese University of Hong Kong, Hong Kong, SAR, China.
3 - Korea Institute of Ocean Science & Technology, Tropical and sub-tropical research center, Jeju-do, 63349, Republic of Korea
4 - Marine Science Institute, University of the Philippines, Diliman, 1101 Quezon City, Philippines.

Table of contents:

Table of contents S1

Figure S2: Schematic representation of the experimental design S2

Figure S3: Diurnal light intensity profiles S3

Figure S4: Average temperature profiles S4

Table S5: Photosynthetic efficiency *Fv/Fm* best-fit model selection S5

Table S6: Mean proportion of total lipid (TL) and total fatty acid (FA) under control conditions S6

Table S7: Mean proportion of total lipid (TL) and total fatty acid (FA) under heat stress conditions S7

Table S8 - S10: Mean proportion of the 21 fatty acids targeted .... S8 - S10

Table S11: Symbiodiniaceae clade proportion S11

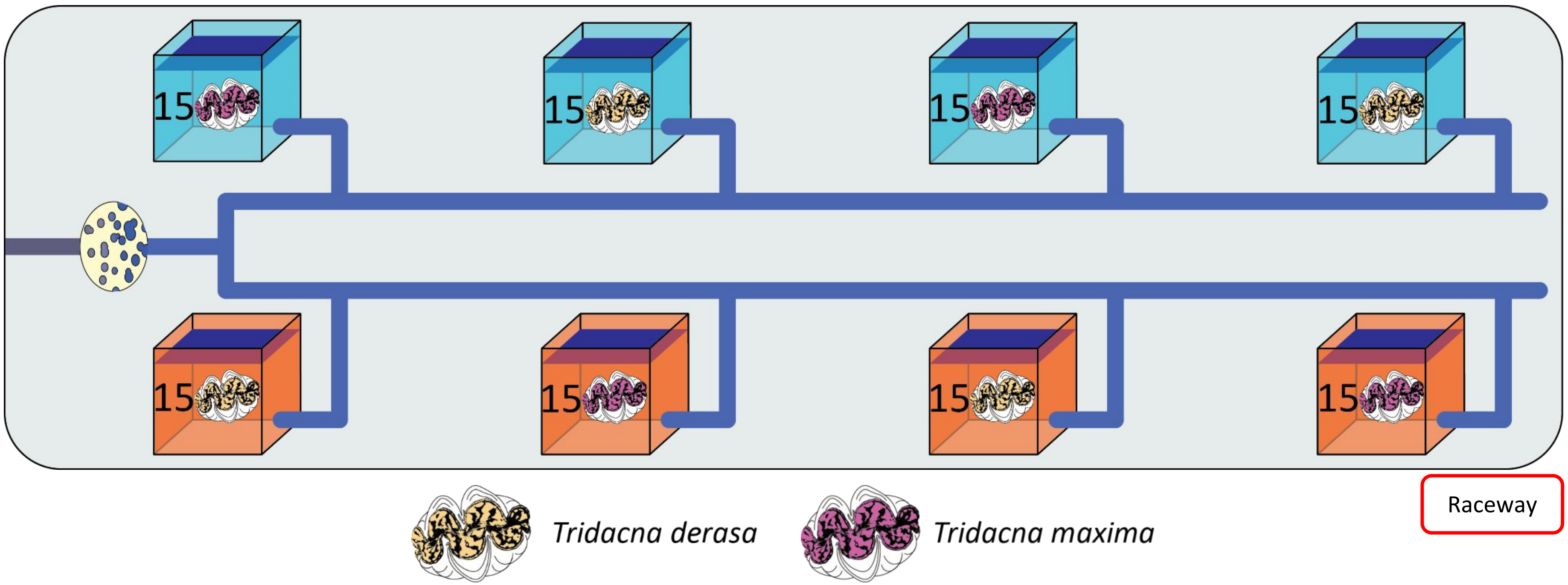

Figure S2: Schematic representation of the experimental design. All aquaria were supplied with seawater pumped directly from the adjacent lagoon (Semirara Marine Hatchery Laboratory, Tabunan Lagoon, Philippines). To minimize sediment load, a coarse filter was used upstream of the aquaria. Four aquaria were maintained at ambient control temperature (Blue), while four others were subjected to thermal stress (Orange). The entire setup took place in a raceway filled with lagoon-temperature seawater.

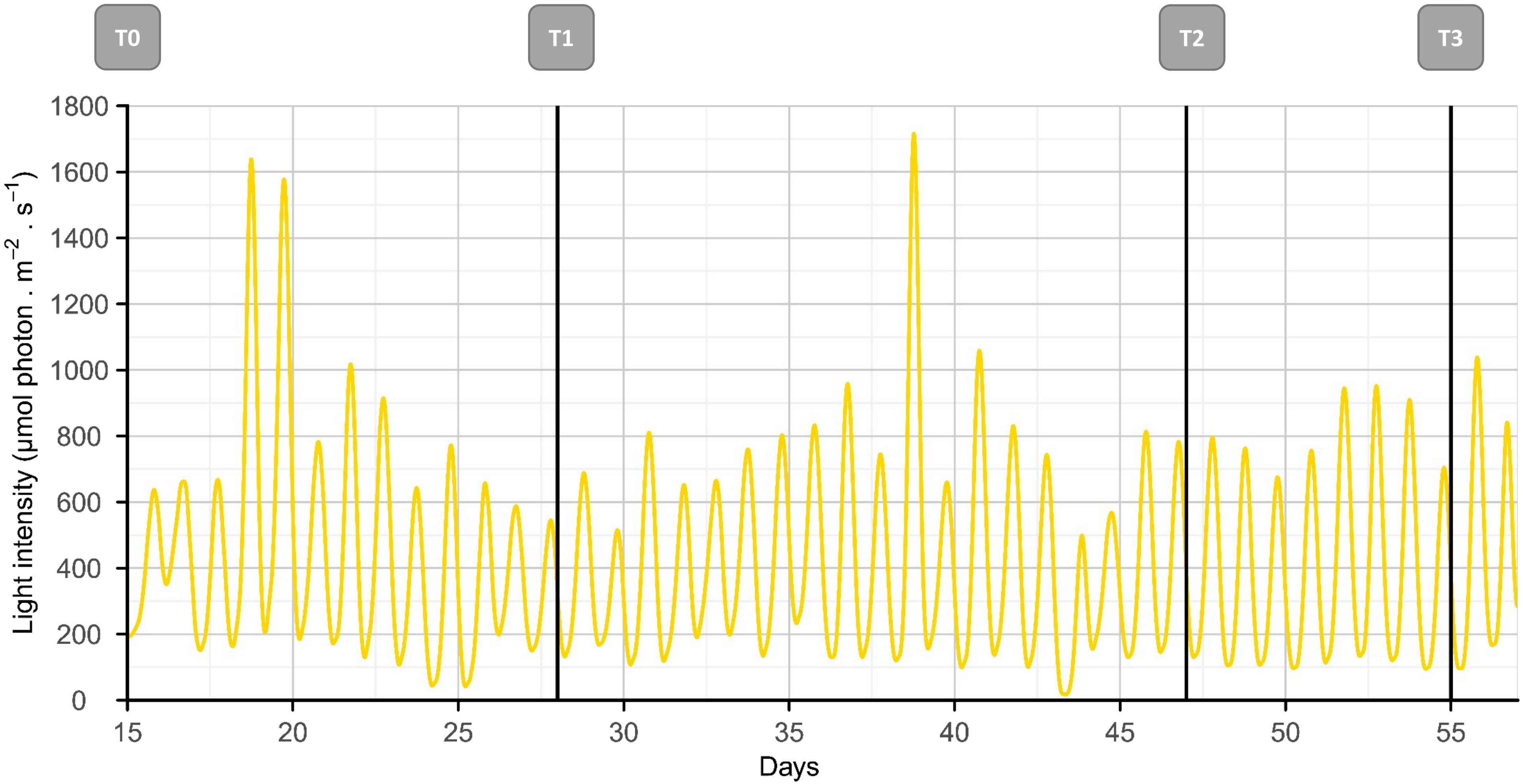

Figure S3: Diurnal light intensity profiles. The moving average light intensity in all aquaria (n=8) shows filtered data including only light measurements between 6:30 a.m. and 5:15 p.m. Labels indicate the four sampling time points. Light intensity remained stable throughout the duration of the experiment.

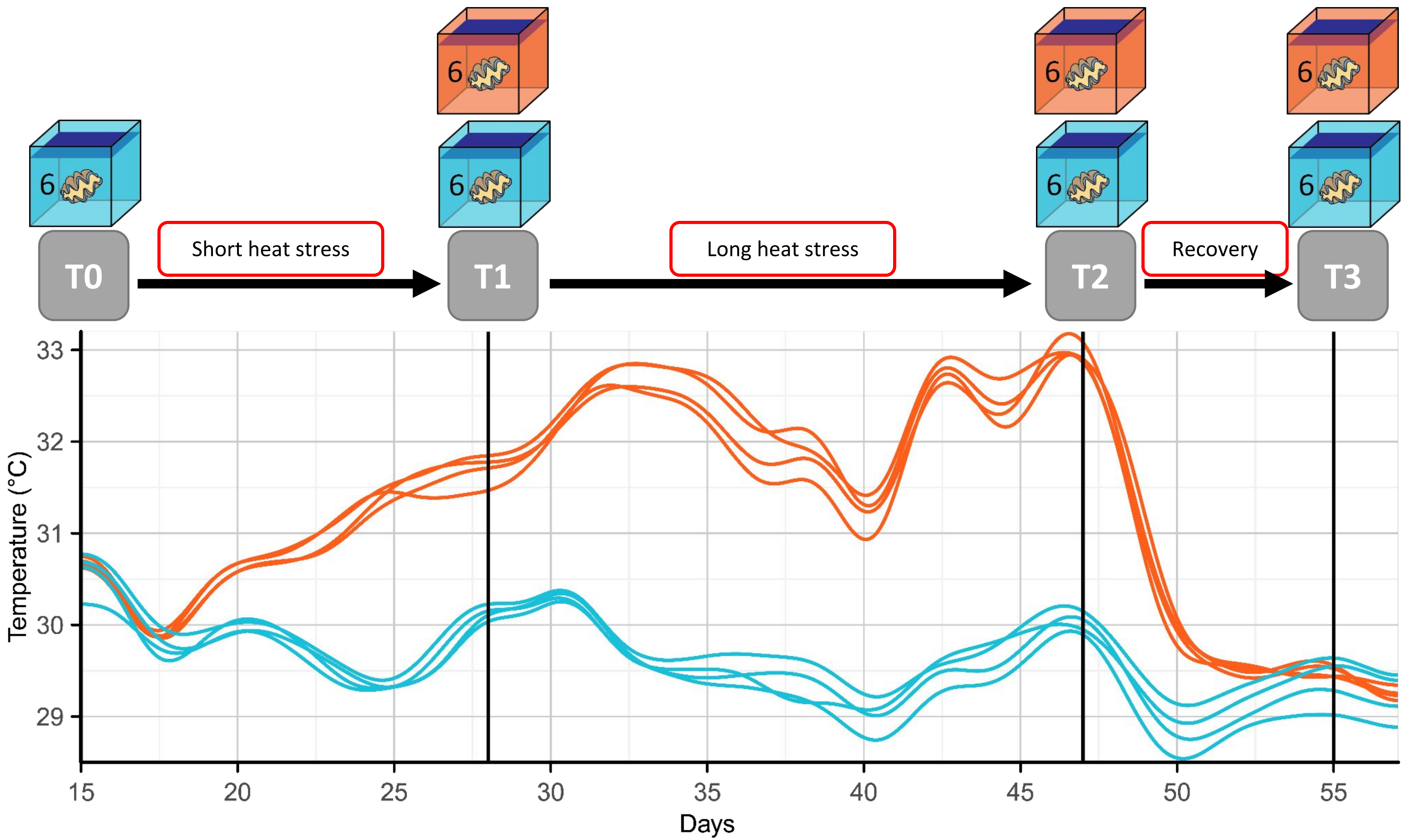

Figure S4: Average temperature profiles (control temperature aquaria: blue; heat stress aquaria: orange). After 15 days of acclimation, six giant clams of each species were sampled as control (T0). For subsequent sampling times, six individuals per species were collected from both heat stress and control temperature aquaria. In order to focus strictly on the physiologic changes caused by temperature, the values obtained from the stressed organisms were normalized to those of the organisms sampled at the same time but from control temperature aquaria (analogue control). A typhoon caused a power outage from day 37 to day 40; however, heat stress aquaria temperatures remained above 31 °C and consistently higher than the control temperature throughout the experiment.

Time period	Species	Treatment	Model	AIC	R^2
T0-T1	*T. derasa*	Control	Linear	383.31	0.3047
			**Log-linear**	**-208.23**	**0.3026**
			Quadratic	380.17	0.3797
		Treatment	Linear	382.23	0.3144
			**Log-linear**	**-209.02**	**0.3137**
			Quadratic	383.80	0.3208
	T. maxima	Control	Linear	420.62	0.1670
			**Log-linear**	**-172.31**	**0.1646**
			Quadratic	413.53	0.3194
		Treatment	Linear	397.91	0.0826
			**Log-linear**	**-194.14**	**0.0821**
			Quadratic	398.29	0.1151
T1-T2	*T. derasa*	Control	Linear	865.68	0.1210
			**Log-linear**	**-451.79**	**0.1212**
			Quadratic	866.00	0.1357
		Treatment	Linear	900.78	0.2076
			**Log-linear**	**-411.53**	**0.2082**
			Quadratic	900.83	0.2229
	T. maxima	Control	Linear	943.39	0.0526
			**Log-linear**	**-377.48**	**0.0523**
			Quadratic	945.10	0.0553
		Treatment	Linear	1051.65	0.6116
			**Log-linear**	**-218.31**	**0.6007**
			Quadratic	1050.03	0.6254
T2-T3	*T. derasa*	Control	Linear	179.96	0.0641
			**Log-linear**	**-82.13**	**0.0646**
			Quadratic	175.20	0.3323
		Treatment	Linear	183.19	0.1935
			**Log-linear**	**-78.81**	**0.1939**
			Quadratic	184.94	0.2034
	T. maxima	Control	Linear	182.14	0.0460
			**Log-linear**	**-81.91**	**0.0459**
			Quadratic	183.97	0.0540
		Treatment	Linear	227.48	0.5876
			**Log-linear**	**-25.93**	**0.5780**
			Quadratic	229.22	0.5929

Table S5: Photosynthetic efficiency *Fv/Fm* best-fit model selection for each species, condition and time period. Three mathematical models were considered to fit the data: linear, log-linear, and quadratic. The best models were selected using the lowest Akaike's Information Criterion (AIC) and are highlighted in bold in the table.

	Control conditions															
	Tridacna derasa								*Tridacna maxima*							
	Host				Symbiont				Host				Symbiont			
	T0	T1	T2	T3	T0	T1	T2	T3	T0	T1	T2	T3	T0	T1	T2	T3
n	6	6	6	6	3	4	4	6	6	6	5	6	5	6	5	6
Dry weight (mg)	11.0± 1.0	11.1± 0.8	10.3± 0.6	10.8± 0.6	5.2± 0.6	4.3± 0.7	4.2± 1.3	5.9± 0.6	11.6± 1.3	10.6± 0.5	10.4± 0.5	10.5± 0.3	4.6± 1.3	5.5± 0.5	6.0± 0.7	5.8± 0.9
Total lipids (% of DW)	41.40± 12.55	34.32± 6.21	46.29± 14.29	35.23± 1.70	54.44± 6.89	77.77± 13.84	65.21± 22.11	53.08± 17.15	31.53± 5.95	33.47± 2.65	35.40± 1.47	36.84± 5.17	38.60± 8.73	35.25± 8.06	31.42± 4.68	33.94± 4.04
Total fatty acid (% of TL)	15.54± 9.13	19.03± 3.22	17.91± 4.42	19.89± 2.75	19.46± 5.70	9.49± 2.10	14.70± 4.11	14.06± 3.72	29.58± 7.73	25.16± 2.16	20.64± 2.03	18.34± 1.85	22.55± 3.82	14.97± 3.51	19.19± 2.00	14.03± 1.96

Table S6: Mean proportions (± sd%) of TL over dried weight (DW) and mean proportions (± sd%) of total FA over TL are reported across all sampling times for both giant clam species maintained under control conditions. No significant differences were observed between sampling times; therefore, values shown represent the averages and standard deviations pooled across all control time points. These values were subsequently used as analogue controls for comparison with stress treatments.

	Stressed condition over average analogue control															
	Tridacna derasa								*Tridacna maxima*							
	Host				Symbiont				Host				Symbiont			
	T0	T1	T2	T3	T0	T1	T2	T3	T0	T1	T2	**T3**	T0	T1	T2	T3
n	5	5	6	6	3	5	5	4	6	6	5	**6**	5	6	6	3
Total lipids (% of DW)	0.00± 30.31	-22.55± 10.21	-8.88± 50.91	-9.19± 5.43	0.00± 12.66	-9.72± 28.55	-18.14± 10.53	3.48± 6.95	0.00± 18.86	-7.76± 25.66	-3.57± 8.60	**78.23± 27.06**	0.00± 22.62	14.10± 23.66	-5.39± 14.86	55.35± 92.27
Total fatty acid (% of TL)	0.00± 58.76	10.42± 14.91	14.02± 75.27	-4.09± 12.49	0.00± 29.31	25.47± 53.06	5.75± 9.76	-6.60± 11.25	0.00± 26.12	-13.37± 15.28	3.84± 6.99	**-54.49± 9.79**	0.00± 16.92	-19.01± 19.48	-9.64± 7.25	-13.71± 51.85

Table S7: Mean proportion (± sd%) of TL over dried weight (DW) and mean proportions (± sd%) of total FA over TL are presented for both giant clam species throughout the experiment. Values represent the ratios of stress treatment measurements to their analogue controls (see supplementary table 2). Significant differences were detected only for *T. maxima* host TL percentages at T3 (after recovery) compared to other time points (statistical results from Kruskal-Wallis and pairwise Wilcoxon tests – supplementary dataset). Changes in total fatty acid relative to TL were largely driven by shifts in TL proportions. As a result, subsequent lipidomic results are reported relative to total fatty acid rather than TL.

	Stressed condition over average analogue control (Panel 1/3)															
	Tridacna derasa								*Tridacna maxima*							
	Host				Symbiont				Host				Symbiont			
	T0	T1	T2	T3	T0	T1	T2	T3	T0	T1	T2	T3	T0	T1	T2	T3
**n**	5	5	6	6	5	5	6	6	6	6	5	6	5	6	6	4
**C14:0**	0.00± 17.05	-15.91± 12.64	3.51± 23.02	-6.97± 11.03	0.00± 31.87	-2.17± 10.22	-6.96± 24.28	-4.04± 19.16	0.00± 26.42	-0.25± 19.85	-33.49± 42.18	-49.41± 15.45	0.00± 33.60	6.95± 34.14	-49.5± 25.35	-45.63± 10.5
**C15:0**	0.00± 15.79	21.04± 3.39	45.38± 16.27	24.77± 13.73	0.00± 27.53	28.95± 12.91	40.83± 31.23	70.62± 29.13	0.00± 72.5	44.36± 19.62	51.97± 23.97	92.2± 43.37	0.00± 19.34	45.21± 11.19	99.06± 45.92	62.95± 17.48
**C16:0**	0.00± 9.38	8.39± 5.60	-0.32± 4.98	-2.77± 3.75	0.00± 13.35	15.31± 7.86	10.51± 9.32	21.10± 10.41	0.00± 8.34	0.44± 5.62	5.51± 5.71	-2.75± 15.31	0.00± 6.65	5.15± 16.92	15.37± 8.26	11.15± 9.11
**C17:0**	0.00± 18.25	24.96± 18.76	28.61± 18.99	14.17± 14.68	0.00± 19.84	18.80± 18.54	37.64± 29.82	35.04± 44.25	0.00± 49.36	26.67± 8.32	47.83± 18.16	38.21± 24.97	0.00± 14.42	25.42± 26.28	98.26± 36.05	54.26± 17.86
**C18:0**	0.00± 16.97	11.29± 5.41	2.95± 3.46	8.52± 10.8	0.00± 30.48	23.06± 12.41	12.48± 21.33	30.04± 17.95	0.00± 17.84	16.54± 9.60	11.46± 10.04	17.39± 16.27	0.00± 22.51	18.93± 35.57	31.28± 21.70	26.38± 12.65
**C20:0**	0.00± 11.17	14.38± 23.65	10.22± 12.02	2.43± 15.13	0.00± 19.39	11.52± 8.24	6.43± 16.43	3.46± 11.02	0.00± 11.06	29.74± 15.67	1.68± 4.71	1.53± 1.86	0.00± 11.20	15.36± 23.37	16.97± 17.96	10.90± 4.85
**C22:0**	0.00± 17.78	23.19± 45.90	23.92± 18.58	4.21± 14.99	0.00± 27.54	11.24± 10.96	4.48± 19.82	3.22± 12.62	0.00± 27.54	67.5± 47.35	22.69± 11.70	4.83± 9.79	0.00± 15.67	39.43± 60.21	43.55± 18.12	4.35± 15.46
**C16:1**	0.00± 44.01	-20.53± 14.82	-26.76± 8.10	-29.92± 7.83	0.00± 69.24	-6.49± 9.14	11.39± 55.72	-21.06± 9.07	0.00± 40.64	-18.35± 15.80	-4.78± 18.23	-36.8± 11.40	0.00± 30.26	-9.52± 27.49	-25.64± 26.79	-30.45± 5.95
**C18:1n-9**	0.00± 14.87	3.24± 2.92	-10.59± 3.57	-10.33± 5.10	0.00± 47.10	-14.11± 14.22	-13.23± 28.33	-31.01± 15.81	0.00± 12.8	17.9± 40.06	4.07± 9.91	7.59± 10.98	0.00± 7.95	19.85± 53.42	9.67± 13.72	14.12± 16.32
**C20:1n-9**	0.00± 23.82	12.91± 7.13	29.5± 11.46	11.15± 7.83	0.00± 30.25	21.91± 15.40	34.61± 34.02	36.23± 43.70	0.00± 47.56	-10.99± 20.09	32.35± 10.69	22.36± 18.35	0.00± 17.45	-3.79± 17.45	88.78± 19.05	33.49± 17.52

	Stressed condition over average analogue control (Panel 2/3)															
	Tridacna derasa								*Tridacna maxima*							
	Host				Symbiont				Host				Symbiont			
	T0	T1	T2	T3	T0	T1	T2	T3	T0	T1	T2	T3	T0	T1	T2	T3
**C22:1n-9**	0.00± 58.72	1.09± 9.89	2.75± 13.46	11.88± 22.29	0.00± 16.62	47.13± 20.55	6.91± 16.96	39.71± 37.81	0.00± 38.92	-14.39± 23.86	23.44± 9.88	25.51± 29.87	0.00± 36.87	7.53± 37.80	72.64± 24.46	28.10± 16.80
**C18:2n-6**	0.00± 44.46	6.70± 10.61	-18.47± 16.12	-34.05± 7.03	0.00± 35.99	13.25± 21.53	-22.29± 15.93	-42.91± 5.55	0.00± 20.06	-23.75± 15.41	-36.62± 23.01	-47.69± 11.92	0.00± 12.24	-22.74± 26.96	-41.54± 17.95	-43.02± 8.25
**C18:3n-3**	0.00± 21.01	-92.99± 1.76	1.41± 8.57	3.49± 13.52	0.00± 34.52	-29.67± 4.55	-8.71± 30.64	-17.75± 32.95	0.00± 7.25	45.46± 83.68	-10.38± 7.13	18.68± 23.16	0.00± 22.95	20.83± 51.31	-14.05± 13.70	0.52± 11.80
**C18:3n-6**	0.00± 99.30	-35.06± 25.99	6.19± 29.70	-16.64± 19.07	0.00± 33.37	9.64± 25.40	-18.66± 28.87	-33.81± 13.12	0.00± 34.63	-47.17± 36.40	-61.69± 10.40	-62.9± 8.75	0.00± 29.01	-38.33± 33.29	-68.11± 12.62	-56.57± 15.46
**C18:4n-3**	0.00± 25.15	-6.13± 8.62	9.11± 10.22	2.45± 21.16	0.00± 20.56	-18.03± 13.60	-12.72± 16.31	-17.24± 15.94	0.00± 39.94	-0.65± 25.12	-42.01± 23.95	-45.44± 15.81	0.00± 11.86	11.73± 108.3	-74.21± 17.80	-62.20± 10.91
**C20:2n-6**	0.00± 12.29	28.40± 9.14	32.42± 10.64	1.14± 10.66	0.00± 19.38	58.24± 11.29	31.66± 16.89	11.57± 23.11	0.00± 27.94	1.06± 14.31	10.25± 6.97	40.1± 31.32	0.00± 16.72	-6.86± 18.90	40.54± 16.40	23.39± 6.46
**C20:3n-6**	0.00± 23.65	-26.69± 4.77	18.94± 43.55	-9.14± 22.78	0.00± 25.02	-29.42± 4.11	-9.54± 3.19	24.53± 30.29	0.00± 11.94	-17.54± 39.29	14.89± 13.64	11.25± 27.27	0.00± 21.78	-18.51± 44.01	49.37± 23.24	28.02± 42.36
**C20:4n-6**	0.00± 21.96	4.75± 14.59	17.00± 8.97	13.31± 8.28	0.00± 35.84	-1.64± 19.09	6.30± 36.31	25.72± 39.85	0.00± 46.9	3.80± 23.32	23.35± 10.25	30.44± 14.58	0.00± 26.50	-9.25± 28.07	54.56± 39.00	24.15± 25.50
**C20:5n-3**	0.00± 10.18	-16.33± 5.35	-17.91± 4.04	0.71± 18.23	0.00± 10.77	-53.04± 10.96	-24.79± 8.77	-19.16± 19.88	0.00± 11.35	27.16± 44.66	-19.76± 3.88	-1.74± 14.21	0.00± 24.91	2.74± 43.15	-31.02± 13.76	-41.45± 14.36
**C22:4n-6**	0.00± 28.95	6.43± 11.61	24.18± 10.44	10.72± 9.11	0.00± 35.95	-18.27± 10.14	20.45± 43.17	19.07± 51.54	0.00± 58.11	-4.35± 32.95	24.19± 13.41	37.25± 23.05	0.00± 18.69	-13.37± 31.81	66.37± 21.06	58.68± 26.58

	Stressed condition over average analogue control (Panel 3/3)															
	Tridacna derasa								*Tridacna maxima*							
	Host				Symbiont				Host				Symbiont			
	T0	T1	T2	T3	T0	T1	T2	T3	T0	T1	T2	T3	T0	T1	T2	T3
**C22:6n-3**	0.00± 12.40	-2.31± 7.58	-13.57± 8.96	-0.21± 9.52	0.00± 9.85	-20.35± 14.75	-13.81± 15.52	-10.36± 7.48	0.00± 4.13	-2.68± 13.12	-13.53± 5.64	-5.08± 22.30	0.00± 10.51	-16.77± 32.85	-32.51± 22.69	-25.66± 13.12
**SFA**	0.00± 11.18	9.51± 3.02	2.43± 3.70	1.29± 5.51	0.00± 18.49	16.57± 8.13	10.33± 11.10	21.7± 11.19	0.00± 2.52	6.11± 3.50	6.85± 3.08	3.51± 15.44	0.00± 9.99	9.92± 20.51	17.70± 10.50	14.73± 9.45
**MUFA**	0.00± 20.04	2.05± 3.43	-6.18± 3.62	-9.26± 4.73	0.00± 52.39	-6.11± 10.27	-1.42± 30.41	-21.15± 13.66	0.00± 7.45	-2.40± 14.33	12.84± 4.28	3.78± 13.16	0.00± 19.08	2.04± 25.18	16.21± 12.52	3.88± 10.88
**PUFA**	0.00± 11.13	-10.41± 2.64	-0.02± 3.2	2.60± 5.45	0.00± 7.94	-18.89± 9.91	-12.00± 11.25	-12.88± 8.23	0.00± 2.88	-5.16± 6.53	-10.18± 2.41	-4.51± 11.70	0.00± 8.49	-14.00± 22.74	-27.29± 12.36	-23.07± 12.19
**SFA:PUFA**	0.00± 17.93	21.91± 6.78	2.52± 6.9	-1.4± 10.26	0.00± 25.23	44.76± 28.16	24.36± 29.58	41.31± 26.15	0.00± 4.78	12.16± 11.29	18.30± 6.42	10.85± 31.88	0.00± 18.94	47.12± 98.34	67.61± 49.44	42.43± 34.56

Table S8 - S10: Mean proportions (± sd%) of each of the 21 fatty acids (FA) targeted over total fatty acid are shown for both giant clam species throughout the experiment. Values represent the ratios of FA concentrations in stressed individuals to their analogue controls. To provide comprehensive data, the table also includes sums of saturated fatty acids (SFA), monounsaturated fatty acids (MUFA), polyunsaturated fatty acids (PUFA), and the SFA:PUFA ratio. Due to the volume of data, statistical analyses (Kruskal-Wallis tests and pairwise Wilcoxon tests with Bonferroni correction) are provided in the supplementary dataset. For each FA, FA class, and SFA:PUFA ratio, significance across sampling times (T0, T1, T2, T3) was assessed using these methods.

Species	***Symbiodinium***	***Cladocopium***	***Durusdinium***
T. derasa	1.7%	92.3% from which 99% are from C93a-C93e-C93n-C93ac	6%
T. maxima	95.9% from which 38% are from A3-A3cj	4.1%	0%

Table S11: Percentage of Symbiodiniaceae associated with both species based on ITS2 metabarcoding data (unpublished data).
